# Myonecrosis in Sickle Cell Anemia—Overlooked and Underdiagnosed

**DOI:** 10.1155/2010/659031

**Published:** 2010-03-11

**Authors:** Nishant Tageja, Marius Racovan, Jason Valent, Jeffrey Zonder

**Affiliations:** ^1^Department of Internal Medicine, Wayne State University/Detroit Medical Center, Detroit, MI 48201, USA; ^2^Division of Rheumatology, Georgetown University, Washington, DC 20057, USA; ^3^Division of Hematology/Oncology, Wayne State University/Karmanos Cancer Institute, Detroit, MI 48201, USA

## Abstract

Medical literature detailing muscular complications of sickle cell anemia is sparse and limited to a few case-reports. Features consistent with myositis and myonecrosis are often overlooked and patients are inadequately treated, leading to unforeseen complications. We report an interesting case of sickle cell myonecrosis and review the existing literature on this subject.

## 1. Introduction

The medical literature on muscular complications of sickle cell anemia is scrubby; while complications such as arthropathy and osteomyelitis [1] are frequently seen and recorded, we could find only a handful of publications detailing muscular problems. Myonecrosis secondary to muscle infarction is a rare complication of sickle cell anemia that is frequently overlooked, resulting in recurrent painful crises and long-term debilitating sequelae for the patient. We report an uncommon case of sickle myonecrosis that developed after recurrent episodes of acute pain and swelling of proximal muscle groups. 

## 2. Case Report

A 28-year-old African American woman with elecrophoretically proved homozygous sickle cell disease and fetal hemoglobin of 1.2% was hospitalized with symptoms of a sickle cell crisis of 2-day duration, which included abdominal, low-back, and shoulder pain. A major facet of this patient's many crises had been tender, swollen proximal muscle groups of the arms. During the preceding three years, the patient had developed firm, indurated deltoid, biceps, and triceps muscles with evolving contractures of both elbows. On third day of hospitalization, while she was being treated with oral opioid analgesics and intravenous hydration, the patient developed acute pain and swelling of the deltoid and triceps muscles. The patient's symptoms of initial generalized crisis had resolved by then. She now had marked tenderness, warmth, and swelling of these muscles in both arms. The rest of the physical examination was unremarkable. Over the next couple of days, the swelling over the muscles increased and was followed by marked limitation in their range of motion. No intramuscular injection had been administered to the affected muscle groups during hospitalization.

**Figure 1:**
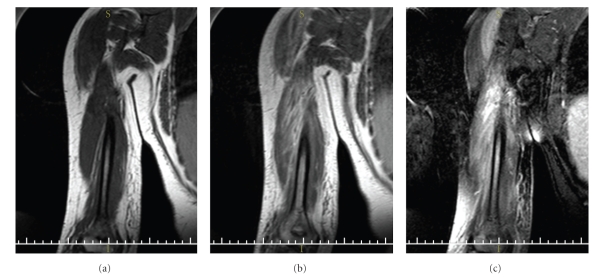
Coronal MRI showing areas of abnormal signal intensity at the inferior aspect of right deltoid muscle and the long and lateral heads of the triceps muscle (b). Please compare to the T1 image without contrast (a). (c) shows postgadolinium enhancement along-with fluid seen in subcutaneous tissue along the lateral aspect of distal right arm.

Laboratory results showed a hematocrit of 27.8% and hemoglobin 9.4 g/dL with normal white blood cell count. Her reticulocyte count was 6.4% with serum lactate dehydrogenase (LDH) 431 U/L. The creatinine phosphokinase level (CPK) rose to 624 U/L. Blood cultures were negative. Radiographs of the shoulders and elbows were normal. MRI scans revealed high signal intensity in the deltoid and triceps muscles, with increased signals in T2-weighed images and postgadolinium enhancement (See Figure 1). Fluid was seen in subcutaneous tissue along the lateral aspect of distal right arm. Absence of rim enhancement ruled out an abscess; no evidence of osteomyelitis was found.

The patient received aggressive physical therapy with oro-systemic pain control and hydration for two weeks. She was pain free at the time of discharge and has not experienced any episodes of myonecrosis or contraction fibrosis in over fifteen months of follow-up.

## 3. Discussion

The clinical presentation of our patient is consistent with a rare and unusual syndrome of sickle cell anaemia consisting of myositis and myonecrosis. Experience of primary care practitioners with sickle cell myonecrosis is limited because of its infrequent occurrence and frequent association with an underlying pain crisis. This clinicopathologic entity usually presents with symmetrical proximal muscle swelling causing severe pain and restriction in movements and characteristic findings on the Magnetic Resonance Imaging (MRI) scan, as detailed below. 

Till date, there have been 13 reported cases of sickle cell myonecrosis (including ours) which are reviewed in Table 1 [2–8]. Most patients are young adults (with the exception of one) and male (9/13), with homozygous sickle cell disease and history of previously treated pain crises. An atypical muscular pain in discrete muscle groups, as the shoulder and thighs, accompanied by swelling and induration is the most common presentation. This pain is out of proportion and different in character when compared to usual pain crises, reflecting a need for careful history-gathering. Three out of four patients have bilateral muscle involvement. Secondary involvement of associated joints (shoulder; knee; quadriceps) is uncommon but can result in decreased range of motion and development of contractures. Other atypical features include development of compartment syndrome (necessitating fasciotomies in 2 patients) and liquefactive necrosis of muscle, which can result in development of a sterile abscess. It is unclear if surgical approach to such abscesses is warranted.

CPK and LDH levels have not been reported in majority of cases. The CPK levels are normal in 3 cases, and elevated in three others (including ours). A rise in LDH has been reported in three patients only, making interpretation of laboratory results challenging.

Muscle biopsy is the gold-standard, but not needed if conclusive MRI-scan evidence is available. Biopsy results are available from 9 reported cases and most commonly reveal acute necrosis of myocytes and occlusion of vessels with sickled red blood cells, accompanied by infiltration of eosinophils and other inflammatory cells. Myonecrosis usually presents with characteristic findings on MRI scan. Necrosis of myocytes causes an alteration in muscle size and shape, which increases signal intensity on T2-weighed images and gadolinium enhancement [9].

If left untreated, progressive necrosis of myocytes can lead to muscle atrophy, fibrosis, and contractures. The optimal management of this syndrome has not yet been determined, but an early diagnosis and involvement of a physical therapist seems crucial in preventing disabling sequelae.

## 4. Conclusions

Myonecrosis is a frequently under-diagnosed complication of sickle cell anemia with disastrous results for the patient if left untreated. There is a need to delineate the risk factors associated with this condition, and develop optimal treatment guidelines to assist the internists/haematologists taking care of patients with sickle cell anemia.

## Figures and Tables

**Table 1:** Reports of Sickle Cell Myonecrosis in medical literature.

Age (years)	Sex	Findings	CPK* (IU/L)	LDH^⋀^ (IU/L)	Muscles involved	Reference
			—			
30	M	Pain swelling warmth tenderness	300	—	B/L deltoids, B/L quadriceps	2
22	M	Pain swelling warmth tenderness	—	—	B/L deltoids, B/L thighs	2
26	F	Pain swelling warmth tenderness	300	—	B/L thigh and knee	3
4	M	Pain swelling tenderness	—	—	Right thigh and knee	3
44	F	Pain swelling indurated contracture	—	—	B/L deltoids	4
36	M	Pain swelling indurated contracture	—	—	B/L quadriceps	4
26	M	Pain swelling induration	—	—	B/L quadriceps	4
30	M	Pain swelling indurated contracture	—	—	B/L thighs	5
42	F	Induration	Normal	—	Left elbow	6
24	M	Pain swelling warmth erythema	2140	592	Left leg	7
24	F	Pain swelling warmth erythema	644	—	B/L thighs	8
28	M	Pain swelling warmth	185	1164	Left thigh	9
28	F	Pain swelling induration	624	431	B/L deltoids, B/L triceps	Our case

*CPK: Creatine Phospho-Kinase; ^⋀^ LDH: Lactate Dehydrogenase.
